# The staging of perihilar cholangiocarcinoma: From anatomy to biology, condition, and beyond

**DOI:** 10.1016/j.iliver.2026.100270

**Published:** 2026-09-18

**Authors:** Yong-Peng Wei, Zi-Jie Tang, Yu-Xiao Chen, Zhuo Wang

**Affiliations:** a Department of Hepatic Surgery, Eastern Hepatobiliary Surgery Hospital, Naval Medical University, Shanghai 200438, China; b Department of Hepatobiliary Surgery, Affiliated Hospital of Nantong University, Nantong 226001, China; c College of Basic Medicine, Naval Medical University, Shanghai 200433, China

**Keywords:** Perihilar cholangiocarcinoma, Hilar cholangiocarcinoma, Staging, ABC score, Patient selection, Artificial intelligence

## Abbreviations

ABC: Anatomy-Biology-Condition
CA19-9: carbohydrate antigen 19-9
pCCA: perihilar cholangiocarcinoma
TRIPOD + AI: Transparent Reporting of a Multivariable Prediction Model for Individual Prognosis or Diagnosis Plus Artificial Intelligence.

Perihilar cholangiocarcinoma (pCCA) remains one of the most challenging malignancies in hepatobiliary surgery. Surgical resection is the only potentially curative treatment, but the 90-day mortality rate has reached 16% in contemporary series,^1^ and the 1-year relapse rate is 40%–65%.^2^ Achieving an R0 margin often necessitates major hepatectomy, biliary reconstruction, and sometimes vascular resection in patients already affected by jaundice, cholangitis, malnutrition, or impaired liver function. Even expert centers cannot completely avoid major morbidity, perioperative mortality, or early recurrence. These competing outcomes raise two linked questions before treatment: whether the risk associated with R0 resection is acceptable and whether upfront resection is likely to be more beneficial than other treatments. Technical resectability may be achieved, but the net surgical benefit may be minimal. Preoperative staging should help identify which patients may benefit from surgery and which require optimization or neoadjuvant treatment before surgery.

Ten Haaft et al. proposed an ABC staging system for resectable pCCA that is efficient but nonetheless merits scrutiny.^3^ They assigned one point each for a tumor size of at least 25 mm, carbohydrate antigen 19-9 (CA19-9) level of at least 500 U/mL, and World Health Organization performance status of at least 1. Patients classified as ABC-3 had a 90-day mortality rate of 21% and median overall survival of 13 months, compared with 6% and 39 months, respectively, in ABC-0. The authors suggested extreme caution in offering resection to patients classified as ABC-3.

The major strength of Ten Haaft et al.'s study was its unusually large cohort of 1307 patients who had undergone resection in 27 centers in 9 countries; thus, the score had statistical power and cross-center credibility. The choice of variables was another strength. All the data can be collected before surgery; thus, the ABC score does not depend on information available only after surgery, in contrast to older systems in which key components, such as nodal status or depth of invasion, are known only after the operation. The statistical methods were rigorous, incorporating multiple imputation, competing-risk analyses, and flexible parametric survival models. Reporting followed the checklist for Transparent Reporting of a Multivariable Prediction Model for Individual Prognosis or Diagnosis Plus Artificial Intelligence (TRIPOD + AI).^4^ The three-item score, however, remains simple enough for use by any multidisciplinary team.

As the time of surgery was considered time 0, the score estimates prognosis after resection rather than outcomes following neoadjuvant or nonoperative treatment. Prognostic and predictive claims are not interchangeable, however.^5^ The same problem affects the cross-trial comparisons used to support recommendations for neoadjuvant or nonsurgical treatment. The survival rates observed among patients who underwent resection in accordance with ABC-3 criteria (13 months) and those who received palliative chemotherapy (12 months) were reported in populations that differed in eligibility, case mix, and time 0; therefore, they cannot quantify the benefit or harm of surgery. Furthermore, the authors reported neither external validation nor an internal–external comparison of results across the 27 centers. Discrimination, calibration, and decision-analytic utility therefore remained undemonstrated, despite the TRIPOD + AI checklist supplied by the authors.^4^ Of further concern, World Health Organization performance status, which accounts for one-third of the score, was unavailable for 59.6% of patients and therefore had to be imputed. The ABC-3 cohort, on which the recommendation for alternative treatment was based, comprised only 6% of the total cohort, and the 5-year survival rate for this subgroup was 11% (confidence interval, 3%–31%). Dichotomizing tumor size at the 25-mm threshold discards prognostic information, and CA19-9 levels are confounded by cholestasis and Lewis-negative phenotypes. As a result, the recommendation to withhold surgery goes beyond the available evidence.

The ABC score is only one of many methods of assessing pCCA (Table 1).^3^^,^^6^, ^7^, ^8^, ^9^ Staging systems for pCCA exist, although the system that dominates clinical practice, the Bismuth–Corlette system, is a purely descriptive anatomic classification and has repeatedly failed to predict resectability or survival.^9^ Its longevity is instructive: what keeps a classification relevant is not primarily prognostic accuracy. Simplicity, availability at the time of decision-making, external validation, guideline endorsement, and, above all, therapeutic consequence are the strengths of a useful classification system. A scheme in which patients are stratified differently without any corresponding change in treatment will rarely be used. Whether the ABC score is adopted will depend on independent external validation, evidence that stage-stratified management improves outcomes, and incorporation into guidelines and multidisciplinary workflows.

**Table 1:** Comparison of established and newly proposed staging/classification systems for perihilar cholangiocarcinoma.

System (year)	Core components	Intended use	Preoperative applicability	Principal limitations
ABC score (2026)^3^	Anatomy (tumor size ≥25 mm), biology (CA19-9 ≥500 U/mL), and condition (WHO PS ≥ 1); 0–3 points	Preoperative prognostic stratification; selection for upfront surgery vs. (neo)adjuvant strategies	Full (three routinely available variables)	Derived for surgical patients only; prognostic rather than predictive findings; external validation pending
AJCC/UICC TNM, 8th ed. (2017)^6^	Tumor extent, nodal status, distant metastasis	Prognostic stratification; standardization of reporting and trials	Partly (node and invasion depth are pathological)	Prone to preoperative understaging; anatomy only; no treatment selection guidance
NCCN resectability criteria (2026)^7^	Imaging-based biliary/vascular involvement, FLR, metastases → resectable vs. unresectable	Surgical eligibility triage	Fully (imaging-based)	Center-dependent interpretation; binary output without graded prognosis; biological processes not mentioned
DeOliveira international staging (2011)^8^	Biliary extent (B), tumor size (T), longitudinal extent (E), vascular (V), nodes (N), metastases (M)	Comprehensive registry–based staging	Partly (several elements best defined pathologically)	Complex; limited uptake outside the registry setting
Bismuth–Corlette (1975)^9^	Longitudinal extent of biliary ductal involvement (types I–IV)	Anatomic description; surgical planning	Fully (imaging/cholangiography)	Vascular invasion, lobar atrophy, and metastases not described; poor predictor of resectability and survival

Abbreviations: ABC, anatomy–biology–condition; CA19-9, carbohydrate antigen 19-9; WHO, World Health Organization; PS, performance status; AJCC, American Joint Committee on Cancer; UICC, Union for International Cancer Control; TNM, tumor–node–metastasis; NCCN, National Comprehensive Cancer Network; FLR, future liver remnant.

A high ABC score does not mean that surgery is unfeasible. For example, a patient with a technically resectable ABC-3 tumor may nonetheless benefit from resection. No data show that surgery produces worse outcomes than nonoperative alternatives in such patients because the 12-month survival rate after palliative chemotherapy reflects different diseases, eligibility criteria, and time 0. In our view, a high score is a reason to prepare more thoroughly rather than less, with preoperative optimization, neoadjuvant therapy inside a protocol, a fully informed discussion, and planned surveillance. Until predictive evidence becomes available, the authors’ reluctance to perform surgery should be viewed as a recommendation rather than a rule. The score can therefore serve as a tool for shared decision-making rather than as a deterrent to the only potentially curative option.

AI may also influence the accuracy and usefulness of staging and risk-assessment approaches. Machine-learning models, radiomics signatures that help predict early recurrence, circulating tumor DNA–informed measures of tumor biology, and multimodal risk engines can capture nonlinear interactions and provide individualized, continuous risk estimates beyond those offered by a three-item score.^2^ The most accurate model, however, is not necessarily the most useful one. Black-box algorithms can be difficult to externally validate, may lack transparency, and can complicate accountability. In contrast, complex models can incorporate numerous variables and be continuously recalibrated, whereas a transparent and auditable tool such as the ABC score can help structure multidisciplinary discussions, trial stratification, and communication with patients. The standard of evaluation should move beyond discrimination alone toward calibration, internal–external validation, and decision-curve net benefit, as recommended under TRIPOD + AI.^4^ The value of a staging system depends on whether its use improves clinical decision-making.

The ABC score advances pCCA staging by incorporating anatomy, biology, and patient condition into a single preoperative framework. Whether it changes practice will depend on external validation, evidence of predictive utility, and acceptance in clinical guidelines. A classification is successful if it leads to useful changes in clinical management. The immediate task is not to determine surgical candidacy by score alone. Rather, the score may help identify patients who need further preparation, warrant particularly careful counseling, or could safely undergo a trial of neoadjuvant or nonoperative treatment before a final treatment decision is made.

### CRediT authorship contribution statement

**Yong-Peng Wei:** Conceptualization, Writing – original draft. **Zi-Jie Tang:** Conceptualization, Writing – original draft. **Yu-Xiao Chen:** Conceptualization. **Zhuo Wang:** Supervision, Writing – review & editing.

### Informed consent

Not applicable.

### Data availability statement

No original data were generated or analyzed for this Editorial.

### Declaration of competing interests

The authors declare that they have no known competing financial interests or personal relationships that could have appeared to influence the work reported in this paper.

### Ethics statements

Not applicable.

### Organ donation

Not applicable.

### Animal treatment

Not applicable.

### Declaration of generative AI and AI-assisted technologies in the manuscript preparation process

During the preparation of this work, the authors used Kimi (Moonshot AI) for language editing and document formatting. The authors reviewed and edited the content as needed and take full responsibility for the content.

### Funding

This work was supported by 2026 Shanghai Health Science Popularization Special Plan (JKKPCX-2026-A09).
